# The Interpersonal Dimension of Pandemic Fear and the Dual-Factor Model of Mental Health: The Role of Coping Strategies

**DOI:** 10.3390/healthcare10020247

**Published:** 2022-01-27

**Authors:** Edita Fino, Denis Mema, Valbona Treska

**Affiliations:** 1 Department of Experimental, Diagnostic and Specialty Medicine, School of Medicine, Alma Mater Studiorum-Bologna University, 40138 Bologna, Italy; 2 Deutsche Gesellschaft für Internationale Zusammenarbeit (GIZ), 1000 Tirana, Albania; denis.mema@giz.de; 3 The Order of Psychologists of Albania (OPA), 1000 Tirana, Albania; presidenti@urdhripsikologut.al

**Keywords:** COVID-19 fear, distress, post-traumatic growth, coping strategies

## Abstract

(1) Background: Current COVID-19 research has mainly focused on negative outcomes associated with fear of the pandemic with the examination of potentially positive outcomes remaining underexplored. Based on the dual-factor model of mental health, which postulates positive and negative dimensions, we assessed the influence of COVID-19 fear on both negative and positive mental health outcomes and examined the mediational role of coping strategies. (2) Methods: A convenience sample of 231 respondents participated in an online survey reporting on measures of pandemic fear (SFS), distress (HADS), post-traumatic growth (PTGI) and individual differences in terms of coping strategies (CSI-SF). (3) Results: Respondents’ main concerns related with the pandemic highlighted the interpersonal and social dimensions implicated in fear of COVID-19. As expected, fear of the pandemic was associated not just with negative but also positive outcomes, while different coping strategies played a role in determining such effects. More specifically, disengagement coping mediated the effects of fear on anxiety and depression, whereas engagement coping was the only mediator of the relationship between COVID-19 fear and post-traumatic growth. (4) Conclusions: Approaches to promote psychological wellbeing in the context of the COVID-19 pandemic should on the one hand be sensitive to the needs of the more vulnerable population groups, while on the other leverage existing resources to harness the potential for growth. Strengthening engagement coping in the context of fears triggered by the pandemic may constitute a valuable target to protect against negative and optimize positive mental health outcomes in the general population.

## 1. Introduction

Pandemics have historically elicited disproportionate levels of fear and distress, arguably due to unique characteristics of infectious disease such as the invisibility, transmissibility and diffusion rate, which make them particularly dreadful compared to other more burdensome conditions [1]. In a pandemic, everyone is a potential target and may become both a victim and a vector. From this perspective, worries about one’s own health are inextricably paired with fear about the health of loved ones and fear of infecting others [2,3]. Furthermore, protection measures commonly endorsed to curb the spread of disease (i.e., physical distancing, lockdown, quarantine) may themselves exacerbate pandemic-related fear, as they disrupt affiliative links and social ties which are crucial to sustaining individuals through adversity [4]. Maintaining positive social interactions with others enhances one’s sense of safety and comfort and acts as a protective factor for health [5]. Thus, differently from other health conditions, fear of a pandemic may be compound by concerns of a more social and interpersonal nature, such as being isolated from the comforting presence of dear ones or being unable to tend to one’s kin in their moment of need, alongside worries about one’s own health and not receiving appropriate healthcare due to overwhelmed facilities [6]. Illustrative in this regard is recent research on the COVID-19 pandemic which highlights the important social and interpersonal implications of the pandemic, painting a picture of generalized psychosocial distress that is sweeping across the world together with the progression of infection [4,7,8].

Notwithstanding such alarming trends, the literature shows that reactions elicited by global pandemics may be far more complex. In a recent study, Wigand, Becker and Steger [9] retrospectively analyzed landmark narratives of three great plagues from different historical eras and found common patterns in peoples’ responses, which seem to construct a much more nuanced account: people felt fear and anxiety but also the pleasure of living for the moment and a sense of strengthened solidarity; there was uncertainty and despair but also compassion and a deeper appreciation of relationships with others and of life more generally [9]. From an evolutionary point of view this is hardly surprising given the different ways human beings cope with threat. Alongside the fight-or-flight response which may represent more proximal and individual reactions, people react to threat through a wide range of affiliative and socially tuned responses such as turning to others for help and caring for the most vulnerable (i.e., the tend-and-befriend response) [5]. A search for meaning is not uncommon and may importantly influence cognitive reappraisal of adversity [10]. In the context of an outbreak, protecting oneself is inextricably linked with protecting others, hence one’s appreciation of social cohesion and caring relationships may deepen. Consequently, when trying to establish potential consequences of pandemic-related concerns and fears on health outcomes, a mixed picture emerges. For instance, fear of the pandemic may lead to the endorsement of restrictive measures (i.e., self-isolation, social distancing, wearing masks) which may contribute to curbing infection rates [11]. On the other hand, if prolonged, social isolation may lead to increasing levels of distress due to the disruption of important affiliative links that crucially support the psychological wellbeing of individuals and communities through periods of hardship [8].

Research on populations going through challenging life experiences—from pandemics [12] and life-threatening diseases such as cancer (for a review see [13]) to natural (for a review see [14]) or humanitarian [15] disasters—has highlighted that only a small proportion develop mental health problems, while a sizeable majority of people are able to adjust and preserve a normal level of functioning [16,17,18]. Moreover, individuals report a sense of growth as a benefit from hardship. Key theorists in this field have coined the term “post-traumatic growth” (PTG) [10] to indicate that the processes involved in counter-acting the negative consequences of challenging life situations may also be involved in leveraging personal resources that may bring one to develop a higher appreciation of life, of relationships with others and of one’s own capacity to persevere amidst adversity.

Individual differences in how people usually cope with difficulty may also play a role in the effects that challenging situations may have on mental health and psychological wellbeing. Coping refers to the ability to enact strategies at the cognitive and behavioral level aimed at counteracting adverse aspects of one’s environment [19] and it may facilitate cognitive processing and functional adjustment when facing adverse events [20,21,22]. For instance, one may cope with a difficult situation by actively engaging in problem solving and trying to find positive solutions. Others may cope by avoiding direct engagement with the stressor altogether and by distracting themselves with other issues. While both are strategies of coping, they differ not only in terms of the approach but also in terms of their effect in downregulating fear and distress. For instance, engaging with the problem is considered a more adaptive strategy compared to disengagement, which has been linked with increased psychological distress and represents a transdiagnostic liability factor for various psychiatric conditions [23].

Based on the recognition that mental health is more than just the absence of psychopathological symptoms [24], when examining the effects of pandemic-related fear both negative outcomes (defined by the presence of mental health problems and clinically relevant symptoms) and positive ones (defined as an optimal way of psychological functioning) should be considered [25,26]. Unfortunately, most research on mental health outcomes associated with COVID-19 has taken a one-sided approach, focusing predominantly on pandemics’ deleterious effects and leaving the potentially positive outcomes unexamined. To date no research has examined the relationship of COVID-19 fear with both negative and positive mental health outcomes while investigating potential mechanisms that may contribute to this relationship. Yet there is a heightened interest in understanding the factors through which pandemics may lead not just to negative but also positive outcomes across population groups and cultural settings, in order to better inform interventions and leverage on existing resources [27].

Two overarching goals guided this study. Firstly, we aimed to assess the personal and interpersonal dimensions of fears related with the pandemic together with self-perceived negative and positive mental health outcomes in a community-based sample. Secondly, we examined whether and how individual differences in coping strategies influence the relationship between fear of COVID-19 and both negative and positive mental health outcomes. We expected the interpersonal and social dimensions to have a central place in compounding pandemic fear and anticipated that this latter would be strongly related not only with heightened distress but also with stress-related growth. Including positive outcomes in the model increases the predictive value of pandemic fear on mental health, which is all the more crucial given that the role of engagement coping strategies was expected to be positively related with positive outcomes and disengagement coping was predicted to be associated with negative mental health outcomes.

## 2. Materials and Methods

A cross-sectional study was conducted from 16 to 30 December 2020 in Albania. Participants (18 to 65 years old) were invited to complete an online questionnaire investigating risk and protective factors for mental health outcomes during the lockdown. The inclusion criteria involved being an adult, residing in the country at the time of data collection, and lacking any major physical and/or psychiatric condition. Recruitment of participants was done through a snowball technique which enabled rapid distribution of the online questionnaire throughout the country. The study protocol and procedure were approved by the Ethics Committee of the OPA (Prot.No.358). All participants provided written informed consent. Measures used in the study were translated and back-translated from English into Albanian by accredited translators in accordance with gold-standard translation practices [28]. Discrepancies were rectified jointly by the research team and independent bilingual individuals with experience in working with healthcare issues.

Pandemic-related fears. Instruments used in other studies to measure fear of COVID-19 range from single items asking respondents to indicate their level of fear on a Likert scale [29], to study specific surveys focusing on specific aspects of pandemic fear [30,31]. For instance, the Fear of COVID-19 scale [30] focuses only on the emotional aspect (i.e., “I am afraid of COVID-19”) and physiological expressions of fear (i.e., “My heart races or palpitates when I think about getting infected”). An evident limitation of this scale is that it leaves out the interpersonal and social aspects which crucially differentiate fear of pandemic from fear of other diseases [1,2,3]. These aspects have been given more weight in other approaches, as for instance in the SARS Fear Scale (SFS) [31] which was originally designed to measure fear of SARS pandemic among healthcare workers given the higher risk of contracting the virus and of infecting others. Considering the limitations of these instruments, in the present research we opted for an adaptation of 8 items from the SARS Fear Scale (SFS) [31] examining areas related to: (a) the perceived risk of infection for self and others; (b) concerns about lack of healthcare due to overwhelmed facilities for self and others; (c) concerns about restriction measures and potential consequences for self and others. In addition, as suggested by recent research [4], 7 items measuring concerns about the psychosocial consequences of prolonged lockdown were added (sample items included “worries about the duration of quarantine”; “worries about the future” and “fear about mine/my family members’ livelihood if quarantine is prolonged”). Respondents were asked to indicate the extent to which they experienced the target concerns during the last month, with response options ranging from 1 (not at all) to 5 (very much/extremely). Responses were summed into a single score, with higher scores indicating higher levels of pandemic fear. The scale demonstrated high internal consistency, with a Cronbach’s α = 0.95. (An English translation of the fear of pandemic scale is provided as Supplementary Material).

Anxiety and Depression. To measure anxiety and depression, we used the Hospital Anxiety and Depression Scale (HADS) [32], which is a 14-item measure that yields two subscales, one for anxiety and one for depression. Each subscale is comprised of 7 items, each taping on anxiety-related (I feel tense or ‘wound up’) and anhedonic (I have lost interest in my appearance) symptoms respectively. Responses are scored on a 0–3 Likert scale with a total score in the range of 0–21 for either anxiety or depression symptoms and are further categorized as: normal 0–7, mild 8–10, moderate 11–14 or severe 15–21 [33]. Both subscales in this study showed an acceptable internal consistency, with a Cronbach’s α of 0.76.

Post-traumatic growth. We used the Post-traumatic Growth Inventory (PTGI) [34] to measure post-traumatic growth which is defined as positive outcomes reported by persons after experiencing traumatic events. The PTGI includes 21 items that load on 5 factors: New Possibilities (I established a new path for my life); Relating to Others (I have a greater sense of closeness with others); Personal Strength (I discovered that I’m stronger than I thought I was); Spiritual Change (I have a better understanding of spiritual matters) and Appreciation of Life (I can better appreciate each day). Respondents are asked to indicate for each item the degree of change occurring in their life as a result of the pandemic. Response options ranged from 0 (I did not experience this change as a result of the pandemic) to 5 (I experienced this change to a very great degree as a result of the crisis). The scale showed good internal consistency, with a Cronbach’s α of 0.87 for Relating to others, 0.85 for New possibilities, 0.83 for Personal strength, 0.63 for Spiritual change and 0.77 for Appreciation of life.

Coping strategies were measured with the Coping Strategies Inventory Short-Form (CSI–SF) [35], which is considered one of the best measures of coping for adults. The CSI-SF is a 16-item scale that generates two overall coping factors, Engagement (I step back from the situation and try to put things into perspective) and Disengagement (I try not to think about the problem), and four secondary factors: Problem-focused engagement; Emotion-focused engagement; Problem-focused disengagement; Emotion-focused disengagement. In the current study, only the Engagement coping (α = 0.81) and Disengagement coping (α = 0.76) factors were used.

**Figure 1:**
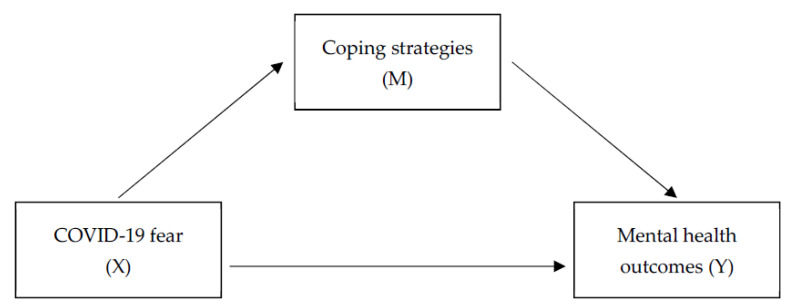
Coping strategies mediate the effects of COVID-19 fear on positive and negative mental health outcomes.

Data analyses were conducted in several stages. First, descriptive statistics were computed to characterize the sample. Additionally, reliability statistics were calculated for scaled variables. To identify potential covariates to include in subsequent analyses, differences between gender and age groups were assessed by ANOVAs and χ2 analyses. Next, a series of mediation analyses were performed with the PROCESS macro for SPSS (Model 4, with 10,000 bootstrap sampling and bias-corrected confidence intervals) [36] to investigate in greater depth the relationship between COVID-19-related worry and both negative and positive mental health outcomes, and to examine the role of coping strategies in this relationship. Figure 1 depicts the mediational model used. The minimum total sample size a priori computed by G-Power software for a medium size effect (f2 = 0.015) for the mediation analysis (with *p* = 0.05 and actual power = 0.95) was 119 participants. All statistical analyses were performed using IBM SPSS Statistics for Windows and the macro-PROCESS (with significance level set at *p* < 0.05).

## 3. Results

### 3.1. Preliminary Analysis

**Figure 2:**
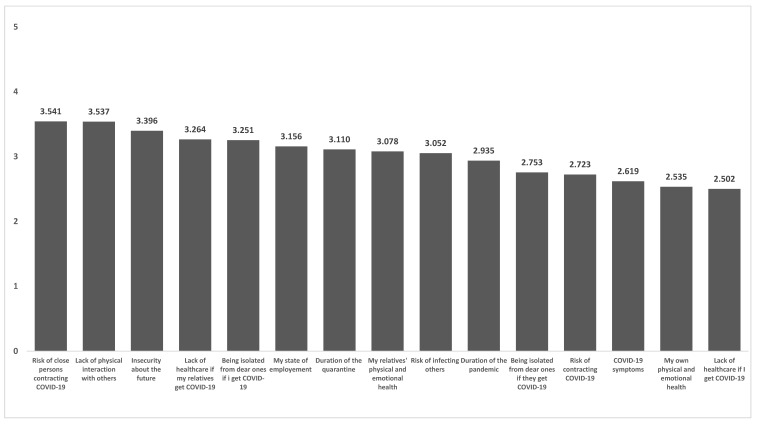
Graphic representation of raw mean scores on fears related with the COVID-19 pandemic reported on a 1 (not at all) to 5 (extremely) response scale by the entire sample (n = 231).

A community-based sample of 231 respondents (73% female, age range 18 to 65) completed the questionnaire. Table 1 shows participant characteristics for the entire sample and separately for males and females. The nature of pandemic fears, rather than being self-focused, revolved around dear ones and the absence of physical contact and social interaction with important others (see Figure 2).

Our participants were most concerned about their loved ones getting infected and lacking appropriate healthcare support if needed. Other major concerns were insecurity about the future, being isolated from loved ones and lack of social interaction with friends and colleagues. Regarding mental health problems, the prevalence of clinically significant symptoms in our sample varied from 16.4% for depression, to 25.1% for anxiety (see Table 1). Significant gender differences emerged for COVID-19 related worries, anxiety and depression with females generally reporting higher levels on raw scores and higher frequency of clinically relevant symptoms, according to recommended cut-off values. Higher levels were also reported by females on positive outcomes such as post-traumatic growth dimensions and also on coping strategies (See Table 1). Gender differences were also found in terms of age with a higher frequency of males among the 25–34 and the 55–65-year-olds. In terms of age group, significant differences were reported for anxiety (F4,227 = 2.654, *p* = 0.034) and disengagement coping strategies (F4,227 = 2.844, *p* = 0.025): those among the 18–24 and the 55–65 year-olds showed the highest levels of anxiety (M _18–24_ = 9.4 ± 3.8; M _25–34_ = 7.6 ± 3.2; M _35–44_ = 8.1 ± 3.0; M _45–54_ = 7.6 ± 2.9; M _55–65_ = 8.7 ± 3.3) and reported highest scores in disengagement coping (M _18–24_ = 8.8 ± 6.5; M _25–34_ = 7.1 ± 5.5; M _35–44_ = 6.8 ± 4.9; M _45–54_ = 5.2 ± 3.6; M _55–65_ = 9.3 ± 4.5).

### 3.2. Pandemic Fear and Negative and Positive Mental Health Outcomes: The Mediating Role of Coping Strategies

The mediation analysis performed on HADS subscales yielded significant results for both anxiety (R2 = 0.50, MSE = 5.656, F4,227 = 72.872, *p* < 0.0001) and depression (R2 = 0.34, MSE = 5.103, F4,227 = 36.760, *p* < 0.0001). That is, the higher the fear, the higher the level of anxiety and depression reported. Disengagement coping was positively related whereas engagement coping was negatively related with both anxiety and depression, as indicated by the beta coefficients (see Table 2 for standardized coefficients of direct, total and indirect effects together with associated 95% confidence intervals). The observed ratios of indirect to total and direct effects indicated a sizeable effect of disengagement coping in mediating the relationship between pandemic fear and distress. The same analysis performed on post-traumatic growth was significant (R2 = 0.24, MSE = 376.173, F4,227 = 23.174, *p* < 0.0001) indicating that fear of COVID-19 was associated with a sense of growth. As expected, a positive relationship was evidenced between engagement coping and post-traumatic growth. Engagement coping was the only significant mediator of the relationship between COVID-19 fear and post-traumatic growth (see Table 2 for standardized coefficients of direct, total and indirect effects together with associated 95% confidence intervals).

## 4. Discussion

As concerns about an upcoming surge in mental health needs mount [27], it is important to continue a conversation on how to protect against negative effects and boost psychological wellbeing during the pandemic in the general population. This study adds to the conversation by advancing the current knowledge on pandemic fear and mental health outcomes by adopting a dual-model of mental health.

Our findings highlight the centrality of the interpersonal and social dimension implicated in pandemic fear causing individuals’ concerns to gravitate on social and affiliative links with others. Respondents in our sample were not as worried about becoming infected themselves as they were about their family members contracting the virus and being unable to access appropriate healthcare due to overwhelmed facilities. The next highest-level concerns regarded lack of social interactions with important others and fear of suffering the illness alone in isolation from dear ones. These data highlight the importance of the social and interpersonal implications of the pandemic, which is in keeping with findings from recent population studies from other countries [37,38]. They also evidence the contribution of protection measures such as lockdown and physical distancing in building up pandemic fear, as suggested by related research [4,8]. Although such measures are certainly necessary to curb the spread of disease, they critically impinge upon one’s adaptive responses to threat, such as turning to important others for support and tending to loved ones in need of care, representing a particularly distressing aspect that accentuates concerns associated with the pandemic [6].

Only a small proportion of the population reported clinically significant symptoms of anxiety and depression; the majority seemed to be adjusting well to the circumstances. These findings are in line with prevalence rates found in recent studies on COVID-19 from countries as diverse as China, Italy, Germany and the US [39,40,41], with data from previous pandemics [2,3] and with the more general literature on natural [14] or human-caused disasters [15].

Also, in keeping with previous evidence [41,42,43], our findings confirm the vulnerability of individuals of female gender in terms of pandemic-related distress: compared to males, female participants in our sample reported significantly higher levels of fear, anxiety and depression. Such differences have been explained by an enhanced stress response which may be biologically wired in females and is putatively further ingrained by gender roles [44,45]. However, women also scored higher on positive outcomes, reporting significantly higher levels than men on all dimensions of post-traumatic growth as well as on coping strategies. This indicates that, notwithstanding a higher vulnerability to stress, women may be more prone to seeing the benefits of a social support system and the importance of caring relationships, which allows them not only to cope better but also to experience a sense of growth in adverse circumstances.

In terms of age differences, our data indicate that both the very young (<24 years) and the older-aged population (55–65 years) may be particularly susceptible to pandemic-related distress. Higher levels of anxiety were reported among these age groups, coupled with a higher tendency to cope through disengagement, which further supports the idea that these population groups may be particularly vulnerable in the face of the COVID-19 pandemic. While older individuals are at higher risk of a poor prognosis if they contract the virus, young adults may suffer more acutely the negative effects of social isolation and lack of physical interaction with others. This finding is in line with recent research indicating both the young and the elderly are disproportionally affected by pandemic-related distress and in need of more support [39,40,43].

The main aim of our study was to evaluate associations of COVID-19-related fear with both negative and positive mental health outcomes and to assess the potential mechanisms that influence this relationship. The most important result of our study is that pandemic fear is associated with both negative and positive outcomes and that this effect is influenced by coping strategies. In line with our expectations, the effects of pandemic fear on anxiety and depression were mediated by disengagement coping, confirming the maladaptive role of avoidance as a coping strategy in response to threat and as a transdiagnostic liability factor for various mental health conditions [23]. Engagement coping, on the other hand, positively mediated the effect of fear on post-traumatic growth, suggesting that cognitively engaging with the situation facilitates functional adjustment and may even lead to a sense of growth. These findings support the notion that the processes involved in coping with adversity may prompt not only pathogenic, but also salutogenic outcomes, putatively through a thorough examination of personal, interpersonal and social resources [46]. Our results importantly advance current knowledge on the role of protective factors for mental health in the context of the current [21,47] and previous [17] global pandemics by examining pathways from pandemic-related fear to both negative and positive outcomes. Notably, the finding that the mediation effects differed between psychological wellbeing outcomes, as well as between coping strategies, is a novel contribution that extends the current literature.

It follows from this evidence that pandemic-related fear is an important factor in determining not just negative but also positive mental health outcomes and that there may be different coping strategies at play in determining such effects. This fits with the dual-factor model of mental health, acknowledging positive and negative dimensions as separate, while correlated amongst them [26]. This evidence suggests that in a pandemic context the level of fear may be high, but it can also be harnessed to increase adaptive behaviors and personal growth while reducing maladaptive responses via engagement coping. Notwithstanding these strengths, some issues might limit the generalizability of our results. For one, our study lacks longitudinal follow-up which would allow the assessment of COVID-19 fear and related consequences as they unfold over time and as a result of the pandemic’s course and protection measures adopted. Secondly, the data collection took place through online platforms which may not be particularly user-friendly for population groups that do not use technological devices, such as those of an older age, and who may have therefore been left out. In addition, the mostly female, on average rather young and well-educated composition of the current sample does not well represent the general population which may further limit the generalizability of these findings. Despite these limitations, our study is the first to show that in a pandemic context, besides fear and distress there can be a potential for growth, and individual variability in coping style can play an important role in this regard.

## 5. Conclusions

As another wave of the COVID-pandemic is ravaging the world in early 2022, questions on how to sustain psychological wellbeing in the general population remain open. Practitioners and policy makers are looking for ways to inform psychological interventions and strengthen resources in efforts to meet mental health needs more effectively [27]. Although the challenges are many, it is important not to overlook existing strengths in the current pandemic landscape. What is evident from the current study is that while negative effects of pandemic-related worry and distress should not be overlooked there is also a potential for growth. Belonging to specific age groups (i.e., the young and the elderly) and being female may increase susceptibility to forms of anxiety and depression associated with the pandemic, especially where combined with the disengagement coping style. On the other hand, having a tendency to cope through engagement seem to protect against such effects and boost personal growth. It follows from this evidence that interventions aimed at promoting community mental health in the context of the COVID-19 pandemic should, on the one hand, be sensitive to the needs of the most vulnerable population groups, while on the other leverage existing resources to harness the potential for growth. This is an important point, considering that coping strategies can be modified and enhanced. Strengthening these psychological resources in the context of fears triggered by the pandemic may constitute a valuable target to protect against negative mental health outcomes and optimize positive ones in the general population.

## Figures and Tables

**Table 1:** Socio-demographic and clinical characteristics of the sample.

	No. (%); Mean (SD)				
	Total Sample (n = 231)	Males (n = 62)	Females (n = 169)	F/χ2	*p*
Age				10.035	0.040
18–24 years	51 (21.1)	8 (12.9)	43 (25.4)		
25–34 years	70 (30.3)	26 (41.9)	44 (26.0)		
35–44 years	63 (27.3)	17 (27.4)	46 (27.2)		
45–54 years	35 (15.2)	6 (9.6)	29 (17.1)		
55–65 years	12 (5.2)	5 (8.0)	7 (4.1)		
Civil status				2.283	0.684
Single	105 (45.5)	26 (41.9)	79 (46.7)		
In a relationship	22 (9.5)	4 (6.4)	18 (10.6)		
Married	95 (41.1)	30 (48.3)	65 (38.4)		
Divorced/widowed	9 (3.9)	2 (3.2)	7 (4.1)		
Education				13.393	0.004
High school	21 (9.7)	12 (19.3)	9 (5.3)		
University	113 (48.9)	32 (51.6)	81 (47.9)		
Post-graduate	97 (42.0)	18 (29.0)	79 (46.7)		
Fear of COVID-19	60.1 (13.1)	55.4 (12.5)	61.8 (13.0)	11.022	0.001
Anxiety (HADS)	8.3 (3.4)	7.5 (2.8)	8.6 (3.5)	4.707	0.031
Cutoff score ≥ 8	58 (25.1)	7 (11.2)	51 (30.1)	8.906	0.012
Depression (HADS)	7.9 (2.7)	7.3 (2.9)	8.2 (2.6)	4.064	0.045
Cutoff score ≥ 8	39 (16.4)	8 (12.9)	31 (18.4)	1.176	0.555
Post-traumatic growth (PTGI)	55.7 (22.4)	47.6 (23.5)	58.8 (21.3)	11.344	0.001
Relationships with others (PTGI)	18.8 (7.8)	16.5 (8.3)	19.6 (7.5)	7.145	0.008
New possibilities (PTGI)	12.2 (5.8)	10.3 (5.6)	12.9 (5.8)	8.733	0.003
Personal strength (PTGI)	11.6 (4.9)	10.1 (5.0)	12.1 (4.7)	6.968	0.009
Spirituality (PTGI)	5.2 (2.8)	4.2 (2.5)	5.5 (2.8)	10.023	0.002
Appreciation for life (PTGI)	8.1 (3.4)	6.6 (3.5)	8.6 (3.2)	16.892	<0.001
Engagement coping (CSI-SF)	18.0 (4.2)	16.8 (4.0)	18.5 (4.3)	7.095	0.008
Disengagement coping (CSI-SF)	7.3 (5.4)	5.9 (4.2)	7.8 (5.8)	5.984	0.015

Note: HADS: Hospital Anxiety and Depression Scale; PTGI: Post-traumatic Growth Index; CSI-SF: Coping Strategies Inventory Short-Form. No. (%) and χ2 statistics are reported for categorical variables (i.e., age, civil status, education, and cutoff scores for anxiety and depression), whereas mean (SD) and F statistics are reported for continuous variables (i.e., post-traumatic growth, anxiety, depression and coping strategies.

**Table 2:** Estimated standardized coefficients for the mediation model of Disengagement and Engagement coping strategies.

	Total Effect				Direct Effect				Indirect Effect (Disengagement Coping)				Indirect Effect (Engagement Coping)			
	*β*	SE	LLCI I	ULC	*β*	SE	LLCI	ULCI	*β*	bootSE	bootLLCI	bootULCI	*β*	bootSE	bootLLCI	bootULCI
Anxiety	**0.559**	0.014	[0.115	0.172]	**0.297**	0.015	[0.045	0.106]	0.334	0.051	[0.237	0.433]	−0.072	0.027	[−0.127	−0.020]
Depression	**0.382**	0.013	[0.054	0.107]	**0.218**	0.015	[0.017	0.075]	0.318	0.055	[0.211	0.428]	−0.155	0.031	[−0.221	−0.098]
PTG	**0.312**	0.108	[0.313	0.738]	**0.282**	0.126	[0.034	0.531]	−0.066	0.040	[−0.147	0.010]	0.211	0.044	[0.131	0.301]

Note: All 95% confidence intervals generated with bias corrected and accelerated bootstrapping (N = 10,000). All findings in bold are significant (*p* < 0.001). Total effect: Effect of COVID-19 fear on anxiety, depression and growth respectively; Direct Effect: Effect of COVID-19 fear on anxiety, depression and growth respectively controlling for coping strategies; Indirect effect: path via coping strategies.

## Data Availability

Data supporting this article shall be made available by reasonable request to the corresponding author.

## Supplementary Materials

The following supporting information can be downloaded at: https://www.mdpi.com/article/10.3390/healthcare10020247/s1; Table S1: The COVID-19 related fears scale.
