# Supplementary material for: The Interpersonal Dimension of Pandemic Fear and the Dual-Factor Model of Mental Health: The Role of Coping Strategies
Source: Healthcare (Basel). 2022 Jan 27;10(2):247. doi: 10.3390/healthcare10020247 (PMC8871641; doi:10.3390/healthcare10020247)
Supplement: Supplementary file 1 [file healthcare-10-00247-s001.zip › healthcare-1555381-supplementary.pdf]

Online Supplementary Material

The COVID-19 related fears scale

The following statements describe a series of thoughts and feelings that might normally arise as a result of the COVID-19 pandemic. Please read each item carefully and indicate the extent to which you have experienced the following during the last couple of weeks on the given scale where 1 = not at all; 2 = a little a bit; 3 = moderately; 4 = a lot; 5 = very much.

1	I am scared about the physical manifestation of symptoms related to COVID-19 virus.	☐	☐	☐	☐	☐
2	I fear I might get infected with COVID-19.	☐	☐	☐	☐	☐
3	I fear that my relatives might get infected with COVID-19.	☐	☐	☐	☐	☐
4	The idea that I might infect other people concerns me.	☐	☐	☐	☐	☐
5	I fear not being able to obtain medical care because of overloaded healthcare facilities.	☐	☐	☐	☐	☐
6	I fear my close relatives not being able to obtain medical healthcare because of overloaded healthcare facilities.	☐	☐	☐	☐	☐
7	I am worried about the pandemic's progress.	☐	☐	☐	☐	☐
8	I am afraid I will be isolated from my close relatives if I contract the virus.	☐	☐	☐	☐	☐
9	I am afraid I will not be able to look after my close relatives if they contract the virus.	☐	☐	☐	☐	☐
10	I am worried about the future if the pandemic persists.	☐	☐	☐	☐	☐
11	I am worried about the duration of quarantine.	☐	☐	☐	☐	☐
12	I am worried about my physical and emotional state if the quarantine period is prolonged.	☐	☐	☐	☐	☐
13	I am worried about the physical and emotional state of my close relatives if the quarantine period is prolonged.	☐	☐	☐	☐	☐
14	I am worried about mine or my family member's employment situation if the quarantine period is prolonged.	☐	☐	☐	☐	☐
15	I am concerned about the lack of physical interaction with friends and colleagues.	☐	☐	☐	☐	☐
